# xCT as a Predictor for Survival in a Population‐Based Cohort of Head and Neck Squamous Cell Carcinoma

**DOI:** 10.1002/cam4.70371

**Published:** 2024-11-02

**Authors:** Linda Nissi, Sanni Tuominen, Johannes Routila, Teemu Huusko, Petra Ketonen, Maria Sundvall, Ilmo Leivo, Heikki Irjala, Heikki Minn, Tove J. Grönroos, Sami Ventelä

**Affiliations:** ^1^ Department of Oncology, and FICAN West Cancer Centre Turku University Hospital and University of Turku Turku Finland; ^2^ Preclinical Imaging Turku PET Centre University of Turku Turku Finland; ^3^ Medicity Research Laboratory University of Turku Turku Finland; ^4^ Department of Otorhinolaryngology—Head and Neck Surgery Turku University Hospital and University of Turku Turku Finland; ^5^ Cancer Research Unit Institute of Biomedicine University of Turku Turku Finland; ^6^ Department of Pathology Turku University Hospital and University of Turku Turku Finland; ^7^ Turku Bioscience Centre University of Turku and Åbo Akademi University Turku Finland

## Abstract

**Background:**

xCT, also known as SLC7A11 (solute carrier Family 7 Member 11), is a cystine/glutamate antiporter protein that mediates regulated cell death and antioxidant defense. The aim of this study was to investigate the effect of xCT on the outcome of patients diagnosed with new head and neck squamous cell carcinoma (HNSCC).

**Methods:**

This retrospective cohort study utilized a population‐based dataset, comprising all patients (*n* = 1033) diagnosed with new HNSCC during 2005–2015 in a population of 697,000 people. All patients (*n* = 585) with a tumor tissue sample available for immunohistochemical (IHC) staining were included. The follow‐up rates were 97% and 81% at 3 and 5 years, respectively. Also, the specificity of the anti‐xCT antibody was validated.

**Results:**

The expression level and prognostic significance of xCT were strongly dependent on tumor location. In oropharyngeal squamous cell carcinoma (OPSCC) patients, xCT expression was a significant prognostic factor for 5‐year overall survival (OAS) (HR: 2.71; 95% CI 1.67–4.39; *p* < 0.001), disease‐specific survival (DSS) (HR: 2.58; 95% CI 1.47–4.54; *p* = 0.001), and disease‐free survival (DFS) (HR: 2.69; 95% CI 1.55–4.64; *p* < 0.001). Five‐year survival rates for OPSCC patients with high and low levels of xCT were OAS 34% versus 62%; DSS 51% versus 73%; DFS 43% versus 73%, respectively. According to a multivariate model adjusted for age, T‐class, nodal positivity, and tobacco consumption, xCT was an independent prognostic factor for 3‐year survival, in which it outperformed p16 IHC. Similar associations were not observed in squamous cell carcinomas of oral cavity or larynx. Regarding treatment modalities, xCT was most predictive in HNSCC patients who received radiotherapy.

**Conclusions:**

High xCT expression was associated with poor prognosis in OPSCC. Our findings suggest that joint analysis of xCT and p16 may add significant value in OPSCC treatment stratification.

## Graphical abstract

xCT, an amino acid transporter that mediates programmed cell death, was evaluated as a biomarker and therapeutic target in head and neck squamous cell carcinoma. In oropharyngeal cancer, xCT was a powerful prognostic factor with potential to outperform p16. The findings also encourage further studies on therapeutic targeting of xCT to overcome radioresistance.

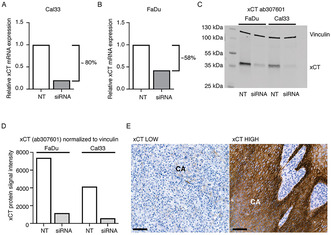

## 1 Introduction

Head and neck squamous cell carcinomas (HNSCC) constitute a heterogeneous group of cancers characterized by a high tendency to relapse [1, 2, 3], especially within 3 years of completing primary treatment [4, 5, 6, 7, 8, 9]. Therapy stratification of HNSCC is still based mainly on clinical features, including TNM staging [2]. Despite extensive research, the expression of p16, a surrogate marker of human papilloma virus (HPV) infection, remains the single established biomarker guiding management of newly diagnosed HNSCC [10].

xCT (system Xc‐), also known as solute carrier Family 7 Member 11 (SLC7A11), has attracted considerable interest in understanding tumor biology and therapeutic targeting. xCT is responsible for transporting cystine into cells and thereby increasing intracellular synthesis of reduced glutathione which plays a central role in the prevention of oxidative stress signaling that regulates cell proliferation and tumor growth [11]. Moreover, xCT‐mediated cystine uptake suppresses ferroptosis, a relatively recently discovered form of iron‐dependent regulated cell death [12, 13, 14], that has been revealed to be a key tumor suppressive mechanism [13, 14, 15]. Ferroptosis dysregulation has also been connected to cancer drug resistance [16, 17]. In addition, ferroptosis plays an important role in radiotherapy‐induced cell death [18, 19], and mediates the synergy between radiotherapy and immunotherapy [20, 21]. Intriguingly, several existing compounds have been demonstrated to act as xCT inhibitors, suggesting the possibility of therapeutic targeting [22, 23]. Therapeutic induction of ferroptosis by, for example, blocking the activity of ferroptosis suppressors has gained interest to increase efficacy of cancer treatments. Ferroptosis induction has been studied as a potential sensitizer for radiotherapy [24, 25, 26]. Moreover, Roh et al. demonstrated that both genetic silencing of the SLC7A11 gene and pharmacological inhibition of xCT by sulfasalazine significantly sensitized cisplatin‐resistant head and neck cancer cells by inducing ferroptosis [27]. In addition, Wang et al. suggested that targeting ferroptosis‐associated metabolism in cancer cells could improve the efficacy of immunotherapy [28].

xCT is demonstrably involved in multiple human carcinomas [29, 30, 31, 32, 33, 34]. In HNSCC, Li et al. suggested that tumor cells might gain uncontrollable proliferation capacity through xCT upregulation to resist ferroptosis [35]. Despite the remarkable interest in xCT, its role in HNSCC has not been fully elucidated due to the small number of studies and partly contradictory results [27, 36, 37, 38]. The challenges underlying the lack of biomarkers in HNSCC include substantial difficulties in translating biomarker findings into clinical practice. This may be partially explained by the bias in inclusion criteria, especially among small retrospective cohorts [10, 39]. To overcome these challenges, we collected a population‐based HNSCC cohort comprising all patients diagnosed with a new HNSCC between the beginning of 2005 and the end of 2015 in Southwest Finland, inhabiting a population of 697,000 people [40]. Consequently, inclusion bias related to health insurance or socioeconomic status can be avoided, and the real‐life heterogeneity in HNSCC patients and their treatment outcomes can be taken account of.

In this study, our aim was to investigate xCT expression in HNSCC in an extensive population‐based setting with an in‐house validated anti‐xCT antibody. The overall goal was to evaluate whether xCT expression predicts overall survival (OAS), disease‐specific survival (DSS), and disease‐free survival (DFS) in HNSCC patients.

## 2 Materials and Methods

### 2.1 Cell Culture, RNA Silencing, Western Blotting, Real‐Time Quantitative PCR, and Immunohistochemistry

The methodology is provided in Supporting Information.

### 2.2 Patients

**FIGURE 1:**
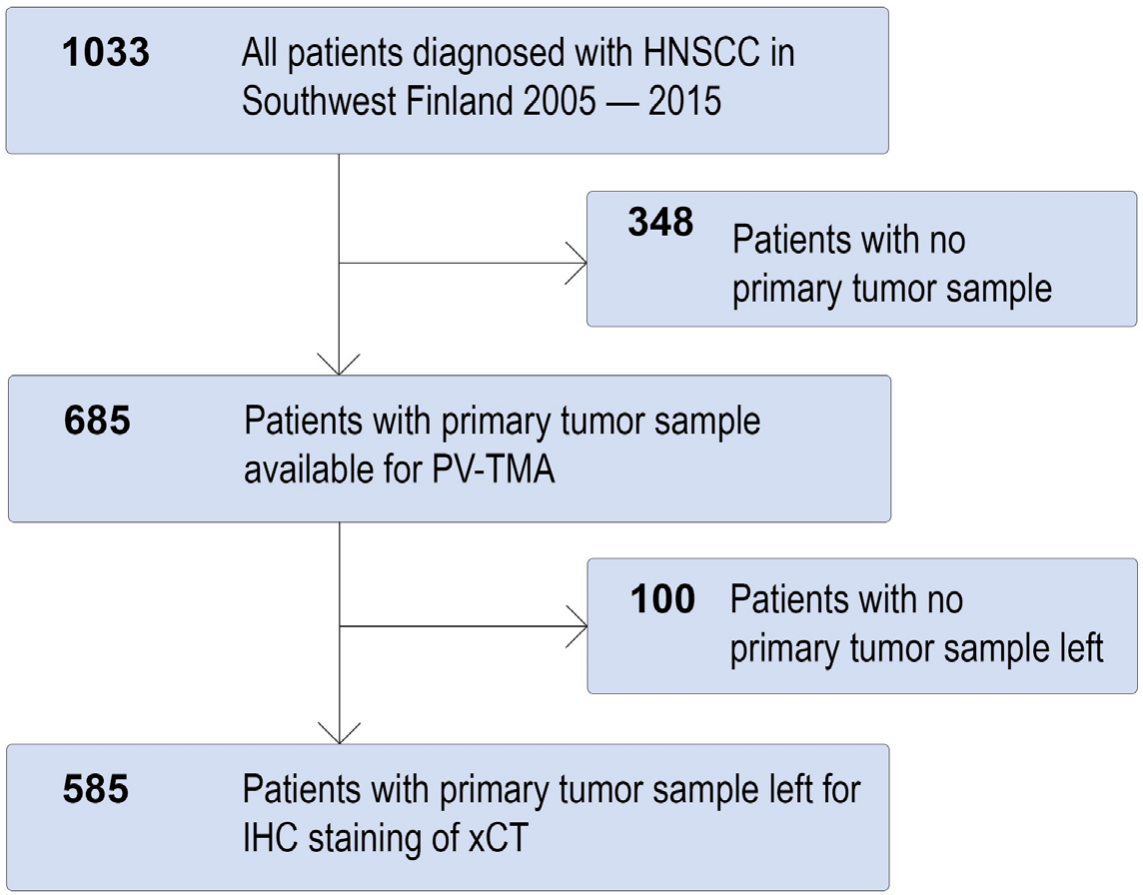
Study population. HNSCC, Head and neck squamous cell carcinoma; IHC, immunohistochemistry; PV‐TMA, population‐validated tissue microarray.

The background patient cohort of this study was formed as described earlier by Mylly et al. [41] and Routila et al. [39, 42] In brief, all new HNSCC patients who were treated between the beginning of 2005 and the end of 2015 in the Southwest Finland tertiary referral center of Turku University Hospital were identified (*n* = 1033). Of these 1033 patients, 685 (66.3%) had a primary tumor sample available for tissue microarray (TMA) analysis. The clinical data of the TMA patients were compared to the background population of all HNSCC patients included in the cohort. The established TMA was considered representative of all new HNSCC patients treated in the Southwest Finland region between 2005 and 2015. Of the 685 patients included in the TMA, 585 (85.4%) got a result from immunohistochemical staining to evaluate xCT expression. Figure 1 illustrates patient inclusion. OAS was defined from the end‐of‐treatment to the end‐of‐follow‐up or death from any cause. DSS was defined from end of treatment to end of follow‐up or death from HNSCC. DFS was defined as the time between the end of treatment and the first disease progression.

### 2.3 Statistical Analysis

All statistical analyses were conducted using SPSS 27 software (SPSS, IBM, Armonk, NY, USA). Logistic regression analysis was used to evaluate differences in the frequency of patients with high and low xCT expression in different patient groups. Survival curves were plotted using the Kaplan–Meier method and compared using the Cox proportional hazards model, which was also applied as a uni‐ and multivariate analysis tool to evaluate the survival effect of xCT. Backward stepwise regression, including all variables in Table 1 (except for site, gender, and treatment), using 3‐year DSS, the likelihood method, and exclusion *p* value of 0.10 in the whole HNSCC cohort was used to identify variables included in the uni‐ and multivariate models. Hazard ratios (HRs) with 95% confidence intervals (CI) and *p* values were reported. *p <* 0.05 were considered significant.

**TABLE 1:** Relationship between xCT expression and clinicopathological parameters. All head and neck squamous cell carcinoma (HNSCC) patients with tissue samples available (*n* = 585) for immunohistochemical staining of xCT were included. Alcohol use was defined as 10 doses or more a week and smoking as daily smoking at the time of diagnosis. T‐class and lymph node metastasis status were determined by pathologic staging.

	Total		Low xCT		High xCT		Logistic regression	*p*
	*n*	%	*n*	%	*n*	%	HR (95% CI)	
Age								
< 65	273	46.7	153	46.9	120	46.3	1	—
≥ 65	312	53.3	173	53.1	139	53.7	1.02 (0.74–1.42)	0.801
Sex								
Female	210	35.9	121	37.1	89	34.4	1	—
Male	375	64.1	205	62.9	170	65.6	1.13 (0.80–1.59)	0.491
T‐class								
T1–2	366	62.8	217	66.8	149	57.8	1	—
T3–4	217	37.2	108	33.2	109	42.2	1.47 (1.05–2.06)	0.026
Unknown 2								
LNM								
N0	326	55.7	173	53.1	153	59.1	1	—
N+	259	44.3	153	46.9	106	40.9	0.78 (0.56–1.09)	0.147
Smoking								
No	299	52.0	196	60.7	103	40.4	1	—
Yes	276	48.0	128	39.3	148	59.6	2.28 (1.63–3.19)	< 0.001
Unknown	10							
Alcohol use								
No	423	74.0	254	78.6	169	67.9	1	—
Yes	149	26.0	169	21.4	80	32.1	1.74 (1.20–2.54)	0.004
Unknown	13							
Site								
Oral cavity	287	49.1	154	47.2	133	51.4	1	—
Oropharynx	138	23.6	85	26.1	53	20.5	0.72 (0.48–1.09)	0.123
Larynx	87	14.9	41	12.6	46	17.8	1.30 (0.80–2.10)	0.286
Hypopharynx	25	4.3	12	3.7	13	5.0	1.25 (0.55–2.84)	0.587
Sinonasal areas^a^	32	5.5	19	5.8	13	5.0	0.79 (0.38–1.67)	0.539
CUP	16	2.7	15	4.6	1	0.4	0.08 (0.01–0.60)	0.014
Treatment								
Surgery	190	32.5	103	31.6	87	33.6	1	—
CRT	105	17.9	59	18.1	46	17.8	0.92 (0.57–1.49)	0.744
RT	44	7.5	25	7.7	19	7.3	0.90 (0.46–1.74)	0.754
Combined^b^	210	35.9	123	37.7	87	33.6	0.84 (0.56–1.24)	0.380
Palliative	36	6.2	16	4.9	20	7.7	1.48 (0.72–3.03)	0.284
p16								
Positive	98	17.2	79	24.9	19	7.5	0.24 (0.14–0.42)	< 0.001
Negative	473	82.8	238	75.1	235	92.5	1	—
Unknown	14							

*Note:* xCT staining scores 0–1 were considered low and scores 2–3 high. Hazard ratios (HR), 95% confidence intervals (95% CI), and statistical significance were calculated using binomial logistic regression analysis.

Abbreviations: CRT, chemoradiotherapy; CUP, cancer of unknown primary; LNM, lymph node metastasis; RT, radiotherapy.

^a^ Sinonasal areas including the nasopharynx.

^b^ Surgery and CRT or RT.

## 3 Results

### 3.1 Anti‐xCT Antibody Validation

**FIGURE 2:**
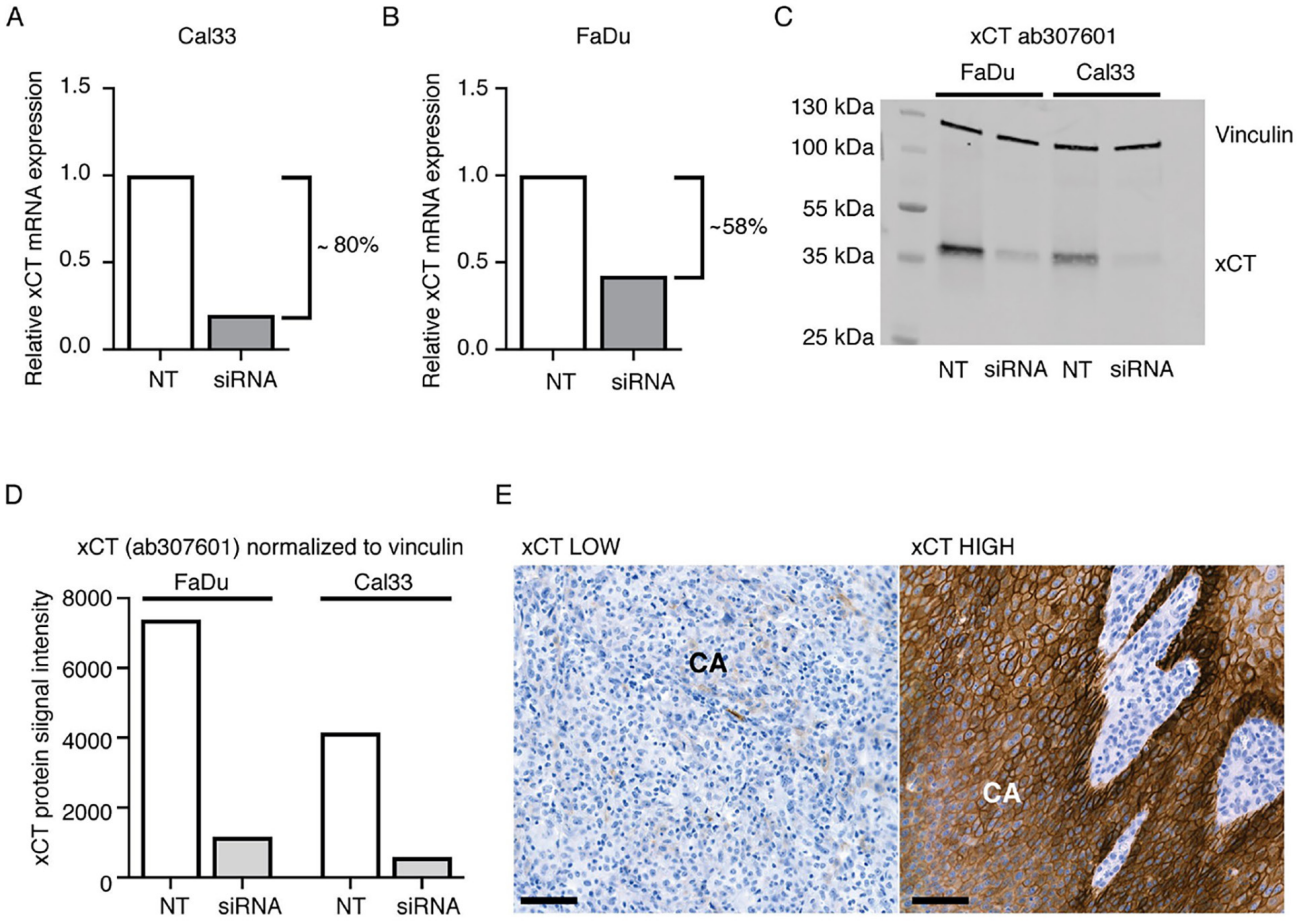
Validation of the anti‐xCT antibody. Specificity of the ab307601 was tested using the siRNA knockdown method followed by real‐time quantitative PCR (RT‐qPCR). Two head and neck squamous cell carcinoma (HNSCC) cell lines, Cal33 and FaDu were used. RNA silencing resulted in downregulation of xCT both at mRNA level (A and B) and in protein signal intensity (C and D) in cells treated with xCT‐targeting siRNA in relation to cells treated with non‐targeting siRNA (NT‐siRNA). Downregulation of xCT in siRNA lanes 2 and 4 (C) demonstrates the specificity of ab307601. Changes in xCT expression were calculated using the delta–delta Ct method. E and F represent xCT immunohistochemistry (IHC) stainings with ab307601 antibody. Staining intensities were classified as low and high, respectively. Black scale bars: 50 μm. CA: carcinoma.

The RNA silencing (siRNA) technique was utilized to evaluate the specificity of three commercial anti‐xCT antibodies (ab307601, ab175186, and CST #12691) in two different HNSCC cell lines, FaDu and Cal33. Using ab307601, successful xCT silencing was confirmed by RT–qPCR, as xCT mRNA expression was reduced by approximately 80% in Cal33 cells and by 58% in FaDu cells compared to that in the corresponding nontargeting (NT) siRNA cells (Figure 2A,B). Western blotting revealed, a clear siRNA‐mediated downregulation of xCT protein expression in both FaDu and Cal33 cells (Figure 2C). No effect on protein expression was detected in cells treated with nontargeting xCT siRNA, demonstrating that ab307601 is specific for xCT detection. Two additional xCT antibodies were unable to show equal specificity against the xCT protein as demonstrated in Figure S1. Therefore, the ab307601 antibody was selected for immunohistochemical stainings. Representative examples of the staining results are presented in Figure 2E.

### 3.2 Locoregional Distribution of xCT in HNSCC and Association to Clinicopathological Features

The characteristics of the patients are presented in Table 1. During follow‐up (median 57 months; range 1–144 months), 242 patients (41.4%) experienced disease recurrence. Of the 585 patients, 32.1% (*n* = 188) died from HNSCC and 20.0% (*n* = 117) died from comorbidities. High expression levels of xCT were observed in 44.3% (*n* = 259) and low levels in 55.7% (*n* = 326) of all HNSCC patients. The proportion of tumors with high xCT expression was greatest in the larynx (52.9%; *n* = 46/87), followed by the hypopharynx (52.0%; *n* = 13/25), the oral cavity (46.3%; *n* = 133/287), and the oropharynx (38.4%; *n* = 53/138). The associations of xCT expression with clinicopathological features is shown in Table 1. A high T‐classification (T3–4) of the primary tumor, daily smoking, and alcohol use of more than 10 units per week at the time of HNSCC diagnosis were associated with high xCT expression.

### 3.3 xCT Has a Prognostic Role in Oropharyngeal Squamous Cell Carcinoma

In the whole HNSCC patient cohort, xCT was not associated with significantly worse 5‐year survival (OAS: HR 1.24; 95% CI 0.98–1.57; *p =* 0.075. DSS: HR1.20; 95% CI 0.90–1.60; *p =* 0.221. DFS: HR 1.23; 95% CI 0.96–1.59; *p* = 0.105), as shown in Figure S2.

**FIGURE 3:**
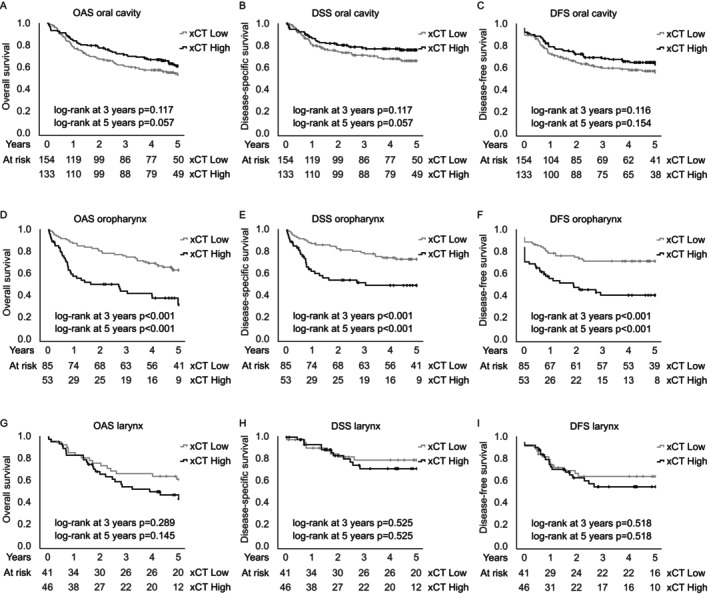
Site‐specific analyses. Prognostic trends with hazard ratios (HR) and 95% confidence intervals (95% CI) for survival in squamous cell carcinoma of the oral cavity (A–C), oropharynx (D–F), and larynx (G–I). The results indicate the diverse role of xCT in tumors of different primary sites. Statistical significance was calculated using Cox proportional hazards model. DFS, disease‐free survival; DSS, disease‐specific survival; OAS, Overall survival.

Thereafter, separate site‐specific analyses were performed for oral cavity, oropharynx, and larynx. As shown in Figure 3, the most significant association with xCT expression and survival was detected in OPSCC patients, in which high xCT was associated with significantly worse survival. Next, uni‐ and multivariate analyses were conducted to further elaborate the prognostic role of xCT. The survival effect of xCT seemed to be most present during the first 3 years. Thus, multivariate analyses for 3‐year survival were conducted. In OPSCC, in a model adjusting for age, T‐class, nodal positivity, and tobacco consumption, high xCT was shown to be an independent prognostic factor for worse 3‐year OAS, DSS, and DFS, as demonstrated in Table 2. Five‐year survival effects of xCT on OAS (HR: 1.57; 95% CI 0.89–2.79, *p =* 0.121) and DSS (HR: 1.78; 95% CI 0.92–3.43; *p =* 0.085) remained nonsignificant in the multivariate model, as presented in Table S1. Nevertheless, the 5‐year survival effect on DFS (HR: 1.95; 95% CI 1.04–3.65; *p =* 0.037) was statistically significant. In oral cavity squamous cell carcinoma (OCSCC), high xCT was not a significant prognostic factor in univariate analyses, as demonstrated in Table S2. However, in a multivariate model adjusting for age, T‐class, and nodal positivity, high xCT was associated with significantly better 3‐year OAS (HR: 0.57; 95% CI 0.38–0.86; *p =* 0.007) and DFS (HR: 0.63; 95% CI 0.42–0.93; *p =* 0.022). In laryngeal squamous cell carcinoma, xCT was not a significant factor in either uni‐ or multivariate analyses, as shown in Table S3.

**TABLE 2:** Uni‐ and multivariate 3‐year survival analysis of oropharyngeal squamous cell carcinoma (OPSCC) patients. Hazard ratios (HR), 95% confidence intervals (CI), and *p* values were reported. Tobacco use was defined as daily smoking at the time of diagnosis.

	Univariate						Multivariate					
	3‐year OAS		3‐year DSS		3‐year DFS		3‐year OAS		3‐year DSS		3‐year DFS	
	HR (95% CI)	*p*	HR (95% CI)	*p*	HR (95% CI)	*p*	HR (95% CI)	*p*	HR (95% CI)	*p*	HR (95% CI)	*p*
Age			1									
< 65	1	—	1.71	—	1	—	1	—	1	—	1	—
≥ 65	1.41 (0.81–2.46)	0.231	(0.94–3.10)	0.080	1.93 (1.12–3.31)	0.017	1.74 (0.98–3.12)	0.060	2.11 (1.14–3.92)	0.018	2.24 (1.27–3.95)	0.005
T‐class												
T1‐2	1	—	1	—	1	—	1	—	1	—	1	—
T3‐4	3.74 (2.05–6.84)	< 0.001	4.45 (2.25–8.84)	< 0.001	3.23 (1.81–5.77)	< 0.001	2.43 (1.29–4.58)	0.006	2.85 (1.38–5.86)	0.005	2.30 (1.23–4.31)	0.010
N‐class												
N0	1	—	1	—	1	—	1	—	1	—	1	—
N+	1.05 (0.58–1.89)	0.875	1.58 (0.78–3.21)	0.201	1.63 (0.86–3.10)	0.138	1.20 (0.65–2.22)	0.562	1.81 (0.88–3.76)	0.110	2.10 (1.06–4.17)	0.033
Tobacco use												
No	1	—	1	—	1	—	1	—	1	—	1	—
Yes	3.86 (2.02–7.39)	< 0.001	3.83 (1.88–7.78)	< 0.001	3.07 (1.65–5.70)	< 0.001	2.49 (1.18–5.27)	0.017	2.43 (1.08–5.49)	0.032	2.21 (1.09–4.48)	0.028
xCT												
Low	1	—	1	—	1	—	1	—	1	—	1	—
High	3.05 (1.75–5.30)	< 0.001	2.87 (1.58–5.23)	< 0.001	2.69 (1.55–4.64)	< 0.001	2.02 (1.06–3.86)	0.033	2.06 (1.03–4.10)	0.041	1.95 (1.04–3.65)	0.037

*Note:* Results from the Cox proportional hazards model.

Abbreviations: DFS, disease‐free survival; DSS, disease‐specific survival; OAS, overall survival.

### 3.4 xCT Outperforms p16 in 3‐Year Survival Prognostication

As the prognostic value of xCT was the highest in OPSCC patients, we first evaluated the benefits of combining p16 and xCT staining. p16 status was available for 99.3% (*n* = 137/138) of OPSCCs [41]. p16‐positive tumors had remarkedly lower xCT expression (HR: 0.24; 95% CI 0.14–0.42; *p* < 0.001) than did their p16‐negative counterparts. Logistic regression was performed to evaluate the correlation between xCT and p16. The logistic regression model was significant (*p* < 0.001) and it explained 34.2% (Nagelkerke *R* [2]) of the variance. p16 stratification did not improve the prognostic resolution of xCT, as demonstrated in Figure S3.

To evaluate whether xCT could bring additional clinical value, we compared it to p16, the only established biomarker in newly diagnosed OPSCC. First, the survival effects of p16 were calculated with a similar multivariate model that was utilized for xCT in the previous chapter. After adjusting for age, T‐class, nodal positivity, and tobacco consumption, the 3‐year survival effects of p16 (OAS: HR 1.99; 95% CI 0.95–4.14; *p* = 0.067. DSS: HR 1.85; 95% CI 0.83–4.11; *p* = 0.131. DFS: HR 1.60; 95% CI 0.78–3.31; *p* = 0.203), presented in Table S4, were weaker than those reported for xCT in Table 2.

**FIGURE 4:**
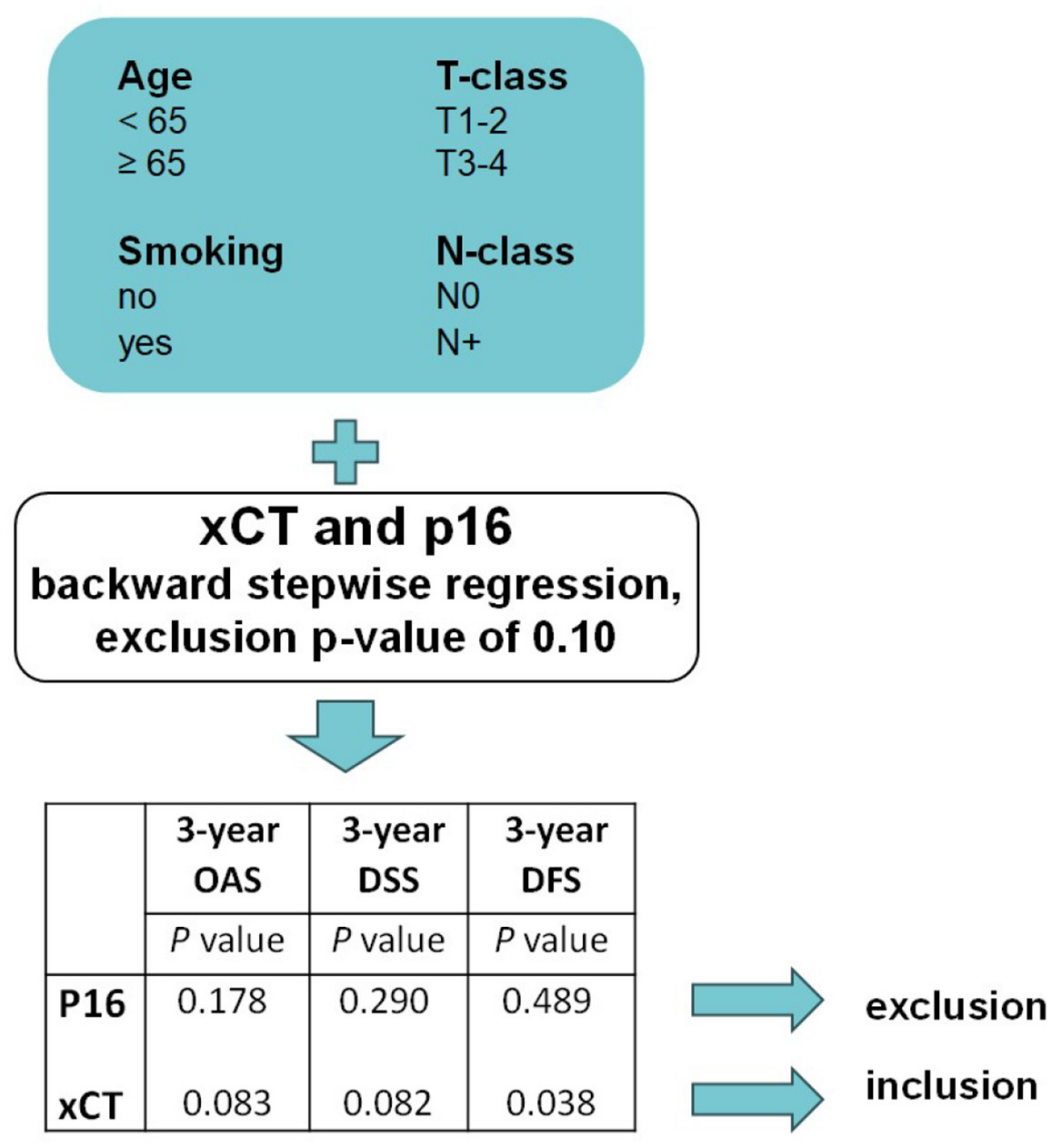
Comparing the independent prognostic value of xCT and p16 in OPSCC patients. xCT (*n* = 138) and p16 (*n* = 137) were entered into the same multivariate model using backward stepwise regression and an exclusion *p* value of 0.10. This resulted in the exclusion of p16 and inclusion of xCT. Smoking was defined as daily smoking at the time of diagnosis. DFS, disease‐free survival; DSS, disease‐specific survival; OAS, overall survival; OPSCC, Oropharyngeal squamous cell carcinoma.

Second, both xCT and p16 were entered into the same multivariate model, which included age, T‐class, nodal positivity, and tobacco consumption, using backward stepwise regression and an exclusion *p* value of 0.10. This procedure, illustrated in Figure 4, resulted in the exclusion of p16, while xCT was included in the model. Finally, p16 and xCT were combined into a product variable with two categories: 1) p16^negative^ and xCT^high^ or 2) any other combination. The product variable, xCT, and p16 were again entered into the previously described multivariate model using backward stepwise regression, as demonstrated in Figure S4. As a result, in 3‐year OAS and DSS, xCT and p16 were excluded, while the product variable was included in the model. In contrast, for 3‐year DFS, the product variable and p16 were excluded, while xCT was included in the model.

In summary, xCT was a better independent prognostic marker for 3‐year survival than p16 in this cohort. However, for OAS and DSS, the best prognostic resolution was achieved when the results from both p16 and xCT stainings were combined. Analogous multivariate models were constructed for 5‐year survival, as described in the Supporting Information, with the same conclusion, suggesting that the product variable best predicts 5‐year OAS and DSS. Nevertheless, xCT alone was the most predictive factor for both 3‐ and 5‐year DFS.

### 3.5 High xCT Expression Predicts Recurrence in HNSCC Patients Receiving Radiotherapy

We also evaluated the prognostic potential of xCT for patients receiving different treatment modalities. For this purpose, patients were divided into two groups based on whether they received radiotherapy as a part of their primary treatment (definitive CRT, definitive RT, or adjuvant therapy). A significant association was observed between high xCT expression and poor 5‐year DFS (HR 1.46; 95% CI 1.01–2.10; *p =* 0.042) in the radiotherapy group, as presented in Figure S5. Moreover, a similar association was not observed in the patient group that underwent surgery only (HR 1.06; 0.72–1.57; *p =* 0.765). Even when adjusted for site, the survival effect of xCT on 5‐year DFS was significant in the radiotherapy group: HR 1.45; 95% CI 1.00–2.10; *p =* 0.049. For a more elaborate analysis, we compared patients who received CRT and RT, as presented in Figure S6. Interestingly, the difference in DFS associated with xCT was present only in the RT group (HR: 2.14; 95% CI: 1.01–4.49; *p =* 0.046). The site‐adjusted survival effect of xCT on DFS in the RT group was as follows: HR 2.28; 95% CI: 1.05–4.92; *p =* 0.037.

## 4 Discussion

xCT/SLC7A11, which promotes redox homeostasis and protects cells from ferroptosis, has been suggested to be a novel prognostic biomarker in HNSCC [35, 36]. In this study, xCT expression was evaluated in an extensive population‐based HNSCC cohort. Our findings suggest that xCT is a prognostic factor particularly in OPSCC, a tumor site not well presented in previous xCT‐related studies. Moreover, xCT outperformed p16 in predicting survival in OPSCC patients in most settings analyzed. We have previously demonstrated that p16 is a prognostic factor in OPSCC patients in the same population‐validated TMA cohort [41]. However, the prognostic role of p16 was shown to decrease markedly when multivariate models were applied. Moreover, several trials have demonstrated a decrease in the prognostic benefit of p16 when treatment deintensification strategies are implemented [43, 44, 45, 46, 47], highlighting the urgent need for complementary biomarkers in OPSCC treatment guidance. In the present study, the best prognostic resolution for OAS and DSS in OPSCC was achieved when p16 and xCT staining were combined, while xCT alone was the strongest biomarker for predicting DFS.

Previously, the role of xCT in HNSCC has been studied mainly in tumors of the oral cavity. Lee et al. [36] have suggested that high xCT predicts posttreatment survival and recurrence in surgically treated patients with oral cavity SCC. In contrast, Toyoda et al. [37] did not find xCT to be a prognostic factor for overall or progression‐free survival in patients with surgically resected tongue cancer. In contrast to our results, Ma et al. [38] reported that xCT can predict overall and recurrence‐free survival in patients with laryngeal SCC. These disparities may be due to differences in the qualities of the antibodies used and the evaluation practices used to measure xCT expression. These are limitations inherent to immunohistochemical techniques and need to be addressed in the present study as well. Problems concerning xCT antibodies, including the debated molecular weight of the protein and batch‐to‐batch fluctuation of antibody specificity, have been previously addressed by Massie et al. [48] We therefore specifically emphasized confirming the specificity of the anti‐xCT antibody by validation via siRNA experiments in two well‐established HNSCC cell lines, FaDu and Cal33.

We confirmed that p16‐negative tumors have higher expression levels of xCT which is consistent with the findings of Hémon et al. [49] Expression of xCT is regulated through a variety of mechanisms, including transcriptional, post‐transcriptional, and post‐translational regulation. Activating transcription factor 4 (ATF4) and nuclear factor erythroid 2‐related factor 2 (NRF2) are two important transcription factors identified to mediate stress‐induced xCT expression [50]. Moreover, p53 that is often mutated in p16‐negative OPSCC, has been demonstrated to regulate the expression of xCT and ferroptosis [14, 51, 52].

Regarding clinicopathological features, we observed that xCT expression was associated with increased T‐class, as reported also in previous studies assessing xCT in HNSCC [36, 37, 38]. In terms of epidemiological risk factors, we found that xCT expression was markedly greater in patients who smoked daily at the time of diagnosis. This finding is in accordance with a previous in vitro study demonstrating that smoking could induce xCT expression in oral cancer cells [53]. However, to our knowledge, this is the first study to demonstrate the association between high xCT expression and smoking in a clinical setting.

Our cohort also enabled us to clarify the predictive ability of xCT in different treatment modalities [41, 54] and we found xCT to be most predictive in HNSCC patients who received radiotherapy. Previously, Lei et al. demonstrated that xCT expression promotes radioresistance through inhibiting ferroptosis [19]. Furthermore, Ye et al. reported the administration of ferroptosis inducers enhanced antitumor effect of radiation [55]. These findings are in line with our results and might explain the remarkably poor survival of patients with high xCT expression in the RT group. We hypothesize that in the CRT group, chemotherapy partially aided in overcoming radioresistance. Thus, xCT might predict the need for concurrent chemotherapy alongside radiation therapy to overcome radioresistance.

The main strength of this study is its population‐based patient and tissue sample collection [41]. All the patients in this cohort were referred to tertiary referral centers and were given the opportunity to receive the most beneficial treatment. Thus, our cohort included unbiased real‐life clinical data with adequate follow‐up time. This study also highlights the important role of different primary tumor sites in HNSCC biomarker studies [56]. However, the variety of sites covered in this study can also be considered a limitation. Although our study on xCT involved the largest number of HNSCC patients thus far, the site‐specific subgroups were relatively small. Therefore, larger, site‐specific and OPSCC‐focused studies are warranted to clarify the role of xCT expression in the heterogeneous disease entity of HNSCC.

In conclusion, we found that the prognostic value of xCT to vary among different primary tumor sites in patients with HNSCC. In OPSCC, high xCT was a more powerful prognostic factor for 3‐year survival than p16. Therefore, xCT might serve as a potential biomarker along with p16 in the treatment stratification of OPSCC patients. Based on its independent prognostic value, xCT could be used to identify OPSCC patients who would likely benefit from treatment intensification. Finally, our results also encourage clinical trials on therapeutic targeting of xCT to overcome radioresistance.

## Author Contributions

**Linda Nissi:** conceptualization (equal), data curation (equal), formal analysis (lead), funding acquisition (equal), investigation (lead), methodology (equal), project administration (equal), software (equal), visualization (equal), writing – original draft (lead), writing – review and editing (equal). **Sanni Tuominen:** conceptualization (equal), formal analysis (equal), investigation (equal), methodology (lead), visualization (equal), writing – review and editing (equal). **Johannes Routila:** data curation (equal), formal analysis (supporting), methodology (equal), writing – review and editing (equal). **Teemu Huusko:** data curation (equal), writing – review and editing (supporting). **Maria Sundvall:** conceptualization (supporting), funding acquisition (supporting), methodology (equal), writing – review and editing (equal). **Petra Ketonen:** investigation (equal), methodology (supporting), writing – review and editing (supporting). **Ilmo Leivo:** investigation (equal), methodology (equal), writing – review and editing (supporting). **Heikki Irjala:** funding acquisition (equal), methodology (supporting), project administration (supporting), supervision (equal), writing – review and editing (equal). **Heikki Minn:** funding acquisition (equal), project administration (supporting), supervision (equal), writing – review and editing (equal). **Tove J. Grönroos:** conceptualization (equal), funding acquisition (equal), methodology (supporting), project administration (equal), resources (equal), supervision (equal), writing – review and editing (equal). **Sami Ventelä:** conceptualization (equal), data curation (supporting), funding acquisition (equal), methodology (supporting), project administration (equal), resources (equal), software (equal), supervision (lead), visualization (equal), writing – review and editing (lead).

## Ethics Statement

The use of human tissue samples was approved by the Finnish National Authority for Medicolegal Affairs (V/39706/2019), the Regional Ethics Committee of Turku University (51/1803/2017), and the Auria Biobank Scientific Board (AB19‐6863). Written informed consent was exempted as permission to use human tissues was granted by the Finnish National Authority for Medicolegal Affairs and the Auria Biobank.

## Conflicts of Interest

The authors declare no conflicts of interest.

## Supporting information


Data S1.

## Data Availability

Data described in the manuscript and analytic code can be made available upon request. Individual patient data cannot be shared due to privacy or ethical restrictions. Requests for deidentified and aggregated research data can be sent to the corresponding author.
