# Supplementary material for: xCT as a Predictor for Survival in a Population‐Based Cohort of Head and Neck Squamous Cell Carcinoma
Source: Cancer Med. 2024 Nov 2;13(21):e70371. doi: 10.1002/cam4.70371 (PMC11530867; doi:10.1002/cam4.70371)
Supplement: Supplementary file 1 — Data S1. [file CAM4-13-e70371-s001.docx]

**Supplement text**

**Cell culture**

FaDu and Cal33 cells were a kind gift from Prof. Anna Dubrovska (OncoRay–National Center for Radiation Research in Oncology, Medizinische Fakultät Dresden, Germany). Cells were cultured in DMEM supplemented with 10% heat-inactivated FBS (E.U.-approved), 1 × GlutaMAX™, 0.5% penicillin-streptomycin (10,000 U/mL), and 1×MEM NEAA (all from Gibco™) in 5% CO_2_.

FaDu and Cal33 cells (10×104 cells/well) were plated on 6-well plates in normal culture medium and allowed to attach overnight. Next day, cells were silenced with Accell Human xCT siRNA SMARTPool or Accell Non-targeting Control Pool (Dharmacon™ Reagents, Horizon Discovery Ltd) according to the manufacturer’s protocol. Briefly, media was replaced with serum-free Accell siRNA Delivery Media (Dharmacon™ Reagents, Horizon Discovery Ltd) with NT or xCT siRNA at a final concentration of 1 µM for 72 h. After silencing, cells were collected in RIPA Lysis and Extraction Buffer supplemented with protease and phosphatase inhibitors (all from Life Technologies Europe BV).

**Western Blot**

Cell samples were lysed in Pierce^TM^ RIPA Buffer (Thermo Scientific) and the protein concentration of the samples was determined with Pierce^TM^ BCA protein assay kit (Thermo Scientific). 4X Laemmli sample buffer (BIO-RAD), 2-mercaptoethanol (Sigma® Life Science) and Pierce^TM^ RIPA Buffer were added to protein extracts. 15 µg of proteins were loaded into precast 4-20% Mini-PROTEAN TGX gels (BIO-RAD) and electrophoresed first at 60V for 20 min, and then at 100V for 120 min. The proteins were transferred onto 0.2 µm PVDF membrane (Trans-Blot Turbo Transfer Pack, BIO-RAD) using the Trans-Blot turbo transfer system (BIO-RAD) at 1.3 A and 25 V for 7 min. The membranes were blocked at RT for 5 min in EveryBlot Blocking Buffer (BIO-RAD) and incubated overnight at 4°C with anti-xCT (1:1000; Abcam Cat# ab307601, RRID not available) or anti-xCT (1:1000; Abcam Cat# ab175186, RRID:AB_2722749) primary antibodies diluted in EveryBlot Blocking Buffer or anti-xCT (1:500; Cell Signaling Technology, Cat# 12691, RRID:AB_2687474) primary antibody diluted in 5% non-fat milk in TBS-T. Vinculin (1:1000; Sigma-Aldrich Cat# V9131, RRID:AB_477629) was used as a loading control. The membranes were washed with TBS-T (5 × 5 min) and then incubated at RT for 1 hour with IRDye® 680RD (1:2000; LI-COR Biosciences, Cat# 926-68072, RRID:AB_10953628) and IRDye® 800CW (1:2000; LI-COR Biosciences, Cat# 926-32213, RRID:AB_621848) fluorescent secondary antibodies diluted in EveryBlot Blocking Buffer with 0.02% SDS. The membranes were washed with TBS-T (6 × 5 min) before fluorescent signal detection with LI-COR Odyssey® CLx Imaging System (LI-COR Biosciences). The fluorescent signals were analyzed with Image Studio Lite 5.2 (LI-COR Biosciences) and the signal intensities were normalized to housekeeping protein.

**Real-time quantitative PCR (RT-qPCR)**

Cell samples were lysed in 1:1 (v/v) RLT buffer (Qiagen) and 96% ethanol. Total RNA was isolated using the RNeasy® Plus Mini Kit (Qiagen) according to the manufacturer’s instructions. RNA was converted into cDNA using Oligo d(T) 18 mRNA Primer (New England BioLabs), dNTP Mix, RiboLock RNAse Inhibitor, RT Buffer, and Maxima Reverse Transcriptase (all from Thermo Scientific). For RT-qPCR, 30 ng of cDNA was used with SsoAdvanced™ Universal SYBR® Green Supermix (Bio-Rad). The primers were hxCT ^1^ and hTBP ^2^. Raw C_t_ values were normalized to the housekeeping gene (TBP) and then to the expression of NT siRNA sample using the delta-delta C_t_ method.

**Immunohistochemistry**

Formalin-fixed and paraffin-embedded (FFPE) samples were acquired from pathology archives through Auria Biobank. Final TMA blocks of duplicate 0.6 mm cores were made in TMA Grand Master (3D Histech) according to annotations on scanned HE slides. Samples of normal liver were included in each block for orientation*.*

xCT expression was determined by immunohistochemical staining with a recombinant monoclonal rabbit antibody (1:3000 dilution, catalog no. ab307601, Abcam). Two independent authors (S.T. and L.N.) analyzed the immunohistochemical staining, and differences were conferred until consensus was reached. xCT expression was scored in a semiquantitative manner based on the intensity of the staining on a scale of 0 – 3. Dichotomous cutoffs were applied for statistical analysis. Immunohistochemical staining of p16 (Roche/Ventana clone E6H4) was performed in a Ventana Bench-Mark XT staining automate (Ventana Medical Systems, Inc., Oro Valley, AZ, USA) in the laboratory of clinical pathology and two independent investigators analyzed the immunohistochemical staining as earlier described by Mylly et al. ^3^.

**Multivariate models comparing xCT and p16 in 5-year survival**

Firstly, effects of p16 on 5-year survival were calculated in a multivariate model adjusting for age, T-class, nodal positivity, and tobacco consumption. The 5-year survival effects of p16 on DSS (HR 1.43; 95%CI 0.67 – 3.02; *p=*0.354) and DFS (HR 1.60; 95%CI 0.78 – 3.31; *p=*0.203) were weaker than those reported for xCT in Supplement Table1. The 5-year survival effects of p16 (HR 1.61; 95% CI 0.85 – 3.06; *p=*0.146) and xCT (HR 1.57; 95% CI 0.89 – 2.79; *p=* 0.121) on OAS were close to equal.

Secondly, both p16 and xCT were entered into the multivariate model, including age, T-class, nodal positivity, and tobacco consumption, using backward stepwise regression and exclusion *p*-value of 0.10. In 5-year OAS, this resulted in the exclusion of p16 (*p=*0.257), followed by xCT (*p=*0.120) in the next step. In 5-year DSS, p16 was excluded first (*p=*0.582). While xCT was included in the model (*p=*0.085) in the model. In 5-year DFS, p16 was excluded (*p=*0.489). While xCT was included in the model (*p=*0.038).

Finally, the product variable of xCT and p16, was entered into the previously described multivariate model along with xCT and p16, using backward stepwise regression. As a result, in 5-year OAS, xCT was excluded first (*p=*0.994), followed by p16 in the next step (*p=*0.631). While the product variable was included in the model (*p=*0.040). In 5-year DSS, p16 was excluded first (*p=*0.964), followed by xCT in the next step (*p=*0.675). While the product variable was included in the model (p=0.059). In 5-year DFS, the product variable was excluded first (*p=*0.765), followed by p16 in the next step (*p=*0.489). While xCT was included in the model (*p=*0.038)


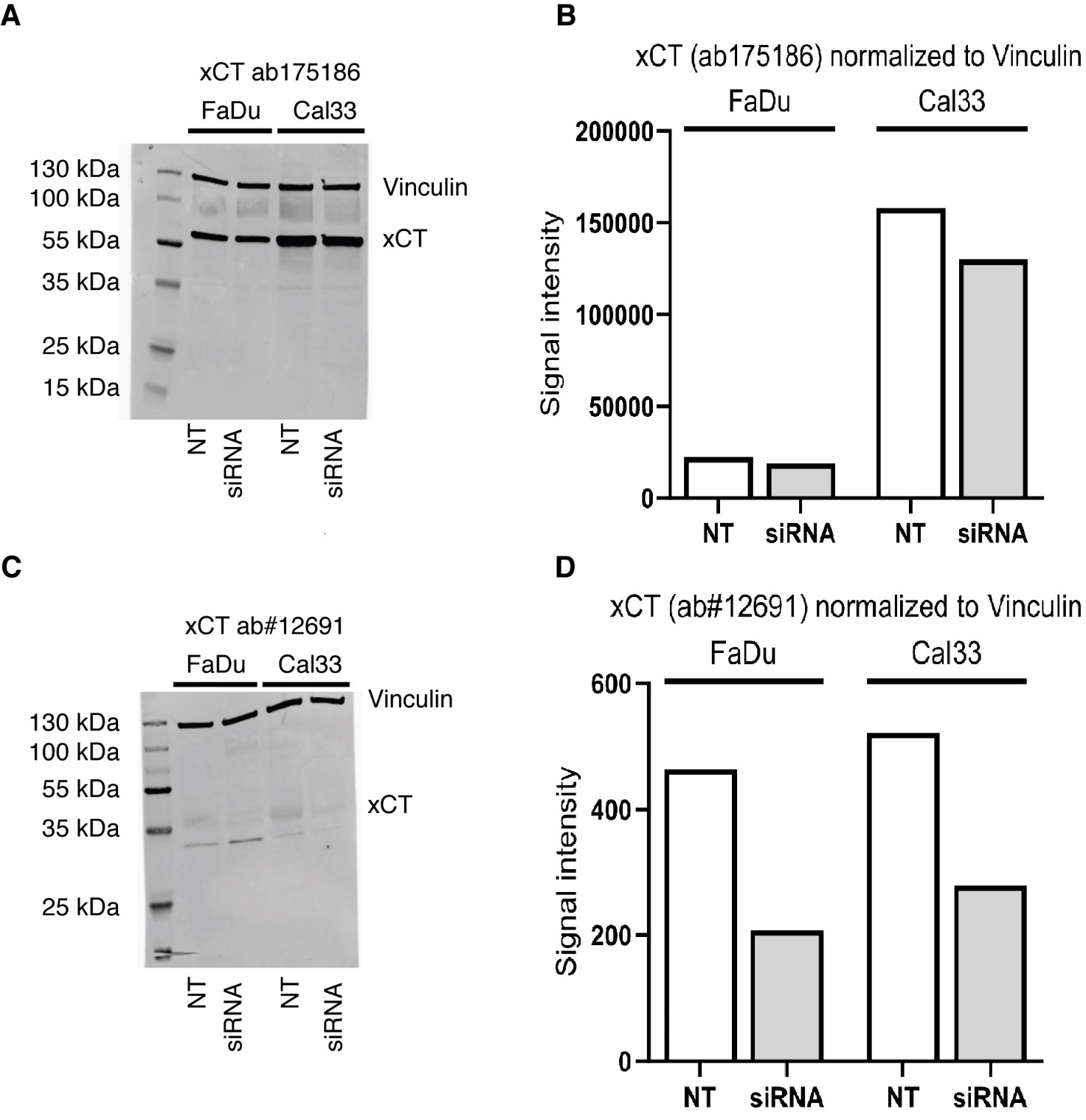


**Supplement Figure 1.** Evaluation of the specificity of two other anti-xCT antibodies. Ab175186 and CST #12691 were tested using the siRNA knockdown method. Two head and neck squamous cell carcinoma (HNSCC) cell lines, FaDu and Cal33 were used. **A** and **C**: Non-targeting (NT) siRNA samples of FaDu and Cal33 were loaded in lanes 1 and 3. xCT-targeting siRNA samples were loaded in lanes 2 and 4, respectively. The ab175186 antibody was unable to distinguish xCT siRNA cells from non-targeting siRNA cells (**B**). The CST #12691 antibody showed almost no signal for xCT in Cal33 cells, whereas the xCT signal was even stronger in xCT-targeting siRNA cells compared to non-targeting siRNA in FaDu cells (**D**).

**
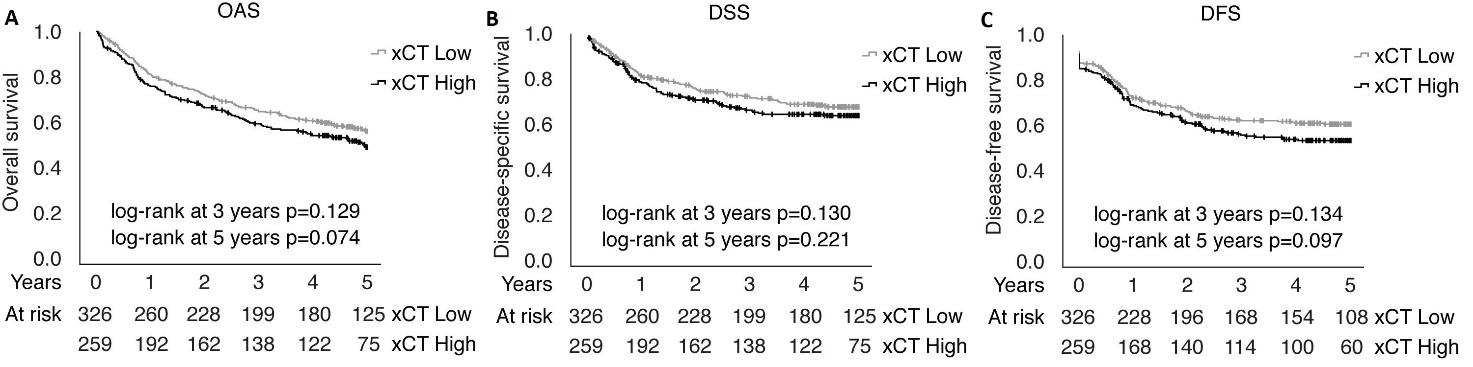
**

**Supplement Figure 2.** Survival in the whole head and neck squamous cell carcinoma (HNSCC) cohort (n=585). Hazard ratios (HR), 95% confidence intervals (95% CI), and statistical significance were calculated using Cox proportional hazard model. Overall survival (OAS); disease-specific survival (DSS); disease-free survival (DFS).


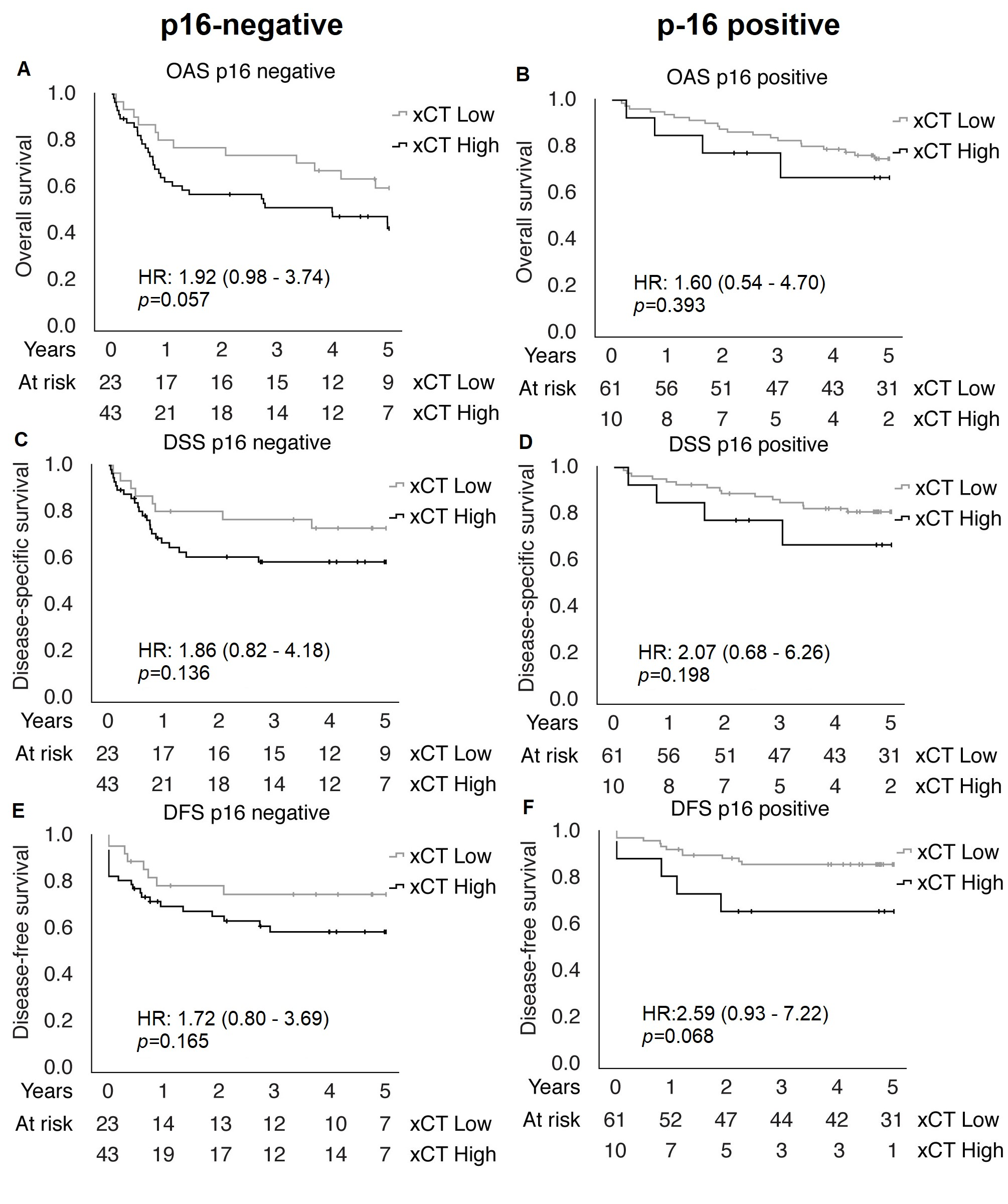


**Supplement Figure 3.** Prognostic trends in oropharyngeal squamous cell carcinoma (OPSCC) according to p16 status. Hazard ratios (HR) and 95% confidence intervals (95% CI) for overall survival (OAS), disease-specific survival (DSS), and disease-free survival (DFS) were reported. Statistical significance was calculated using Cox proportional hazard model.


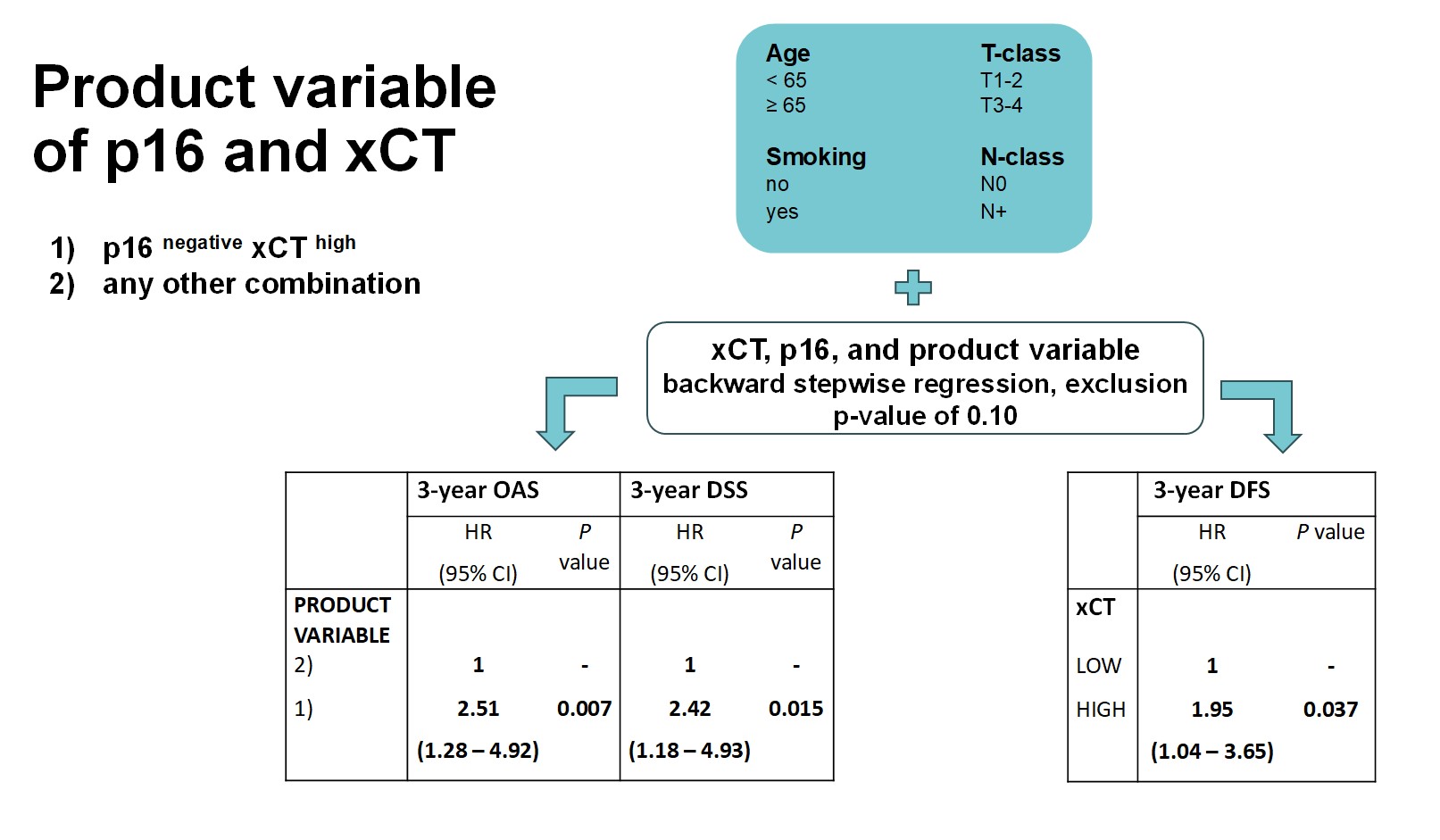


**Supplement Figure 4.** Comparing the independent prognostic value of xCT, p16 and their product variable in oropharyngeal squamous cell carcinoma (OPSCC). p16 and xCT were combined into a product variable with two categories. The product variable, xCT, and p16 were entered into the multivariate model using backward stepwise regression and an exclusion p -value of 0.10. As a result, in 3-year OAS and DSS p16 and xCT were excluded, while the product variable was included in the model. In contrast, for 3-year DFS, the product variable and p16 were excluded and xCT was included in the model. Smoking was defined as daily smoking at the time of diagnosis. Overall survival (OAS); disease-specific survival (DSS); disease-free survival (DFS).


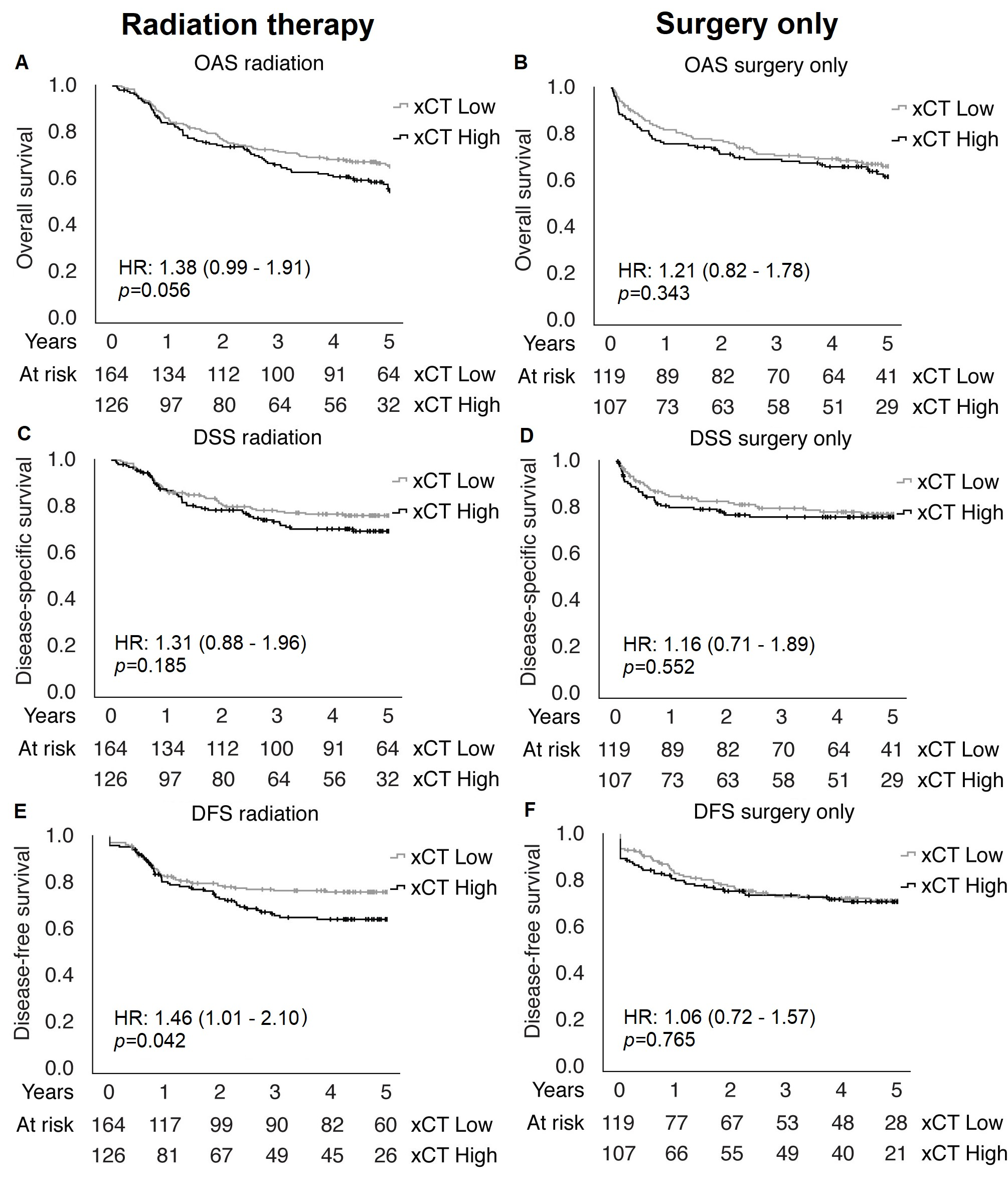


**Supplement Figure 5.** Prognostic trends according to treatment type. The radiation therapy group is defined by patients who received radiation therapy as a part of their treatment (definitive radiation therapy, definitive chemoradiation therapy, or in combination to surgery). Hazard ratios (HR), 95% confidence intervals (95% CI), and statistical significance were calculated using Cox proportional hazards model. Overall survival (OAS); disease-specific survival (DSS); disease-free survival (DFS).


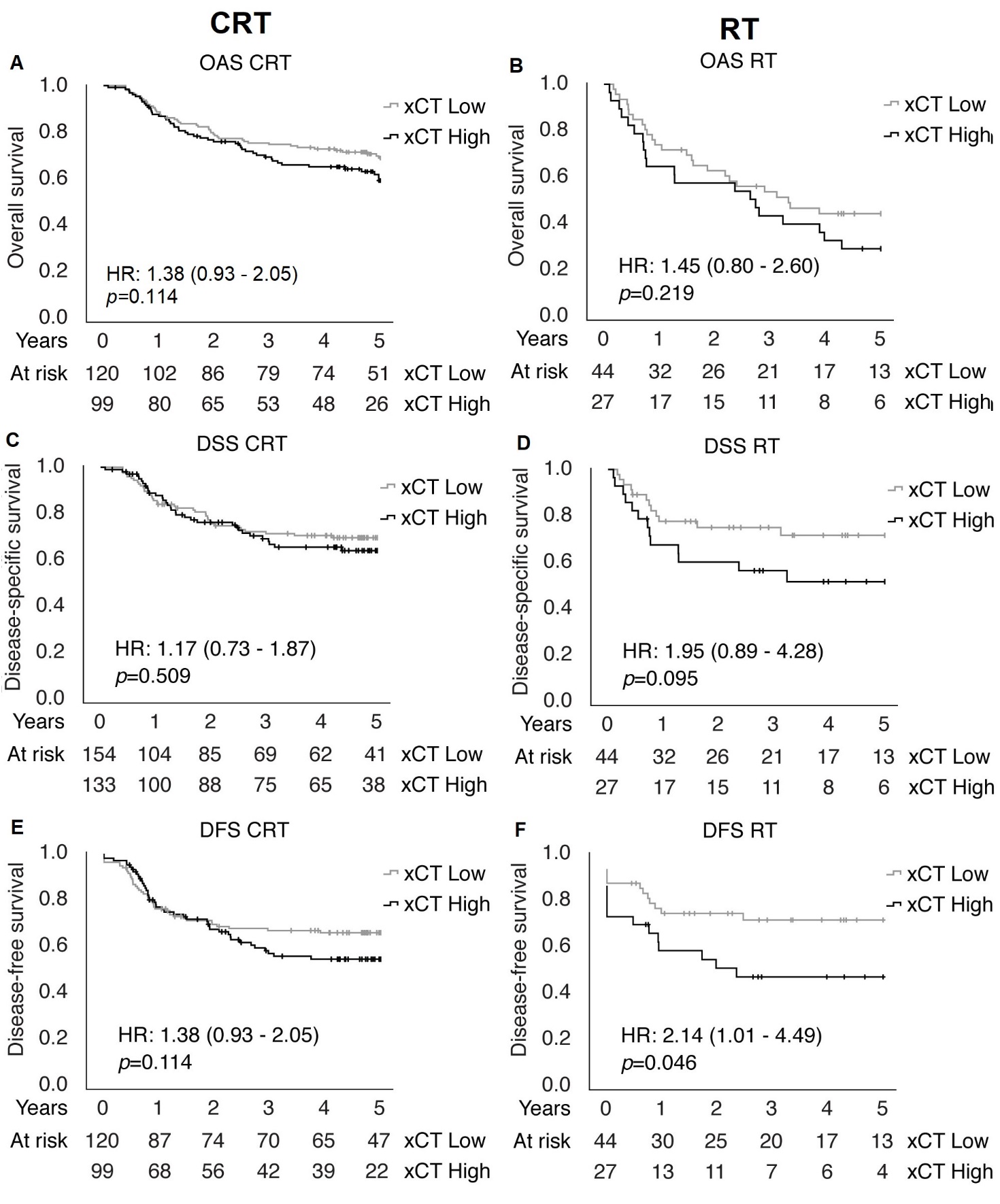


**Supplement Figure 6.** Survival in patients receiving chemoradiation therapy (CRT) and radiation therapy (RT) as a part of their first-line cancer treatment. Hazard ratios (HR), 95% confidence intervals (95% CI), and statistical significance were calculated using Cox proportional hazard model. Overall survival (OAS); disease-specific survival (DSS); disease-free survival (DFS).

**Supplement Table 1.** Multivariate 5-year survival analysis of oropharyngeal squamous cell carcinoma (OPSCC) patients. Hazard ratios (HR), 95% confidence intervals (CI), and *p* values were reported. Statistical significance was calculated using the Cox proportional hazards model. Tobacco use was defined as daily smoking at the time of diagnosis.

|  | **5-year OAS** |  | **5-year DSS** |  | **5-year DFS** |  |
| --- | --- | --- | --- | --- | --- | --- |
|  | Survival effect  HR (95% CI) | *P* value | Survival effect  HR (95% CI) | *P* value | Survival effect  HR (95% CI) | *P* value |
| **Age**  < 65  ≥ 65 | 1  1.79  (1.08 – 2.97) | -  0.025 | 1  2.01  (1.12 – 3.60) | -  0.019 | 1  2.24  (1.27 – 3.95) | -  0.005 |
| **T-class** |  |  |  |  |  |  |
| T1-2 | 1 | - | 1 | - | 1 | - |
| T3-4 | 2.97  (1.69 – 5.22) | < 0.001 | 3.66  (1.81 – 7.44) | < 0.001 | 2.30  (1.23 – 4.31) | 0.010 |
| **N-class** |  |  |  |  |  |  |
| N0 | 1 | - | 1 | - | 1 | - |
| N+ | 1.22  (0.70 – 2.12) | 0.476 | 1.79  (0.89 – 3.59) | 0.097 | 2.10  (1.06 – 4.17) | 0.033 |
| **Tobacco use** |  |  |  |  |  |  |
| No | 1 | - | 1 | - | 1 | - |
| Yes | 2.91  (1.51 – 5.60) | 0.001 | 2.31  (1.09 – 4.90) | 0.030 | 2.21  (1.09 – 4.48) | 0.028 |
| **xCT** |  |  |  |  |  |  |
| low | 1 | - | 1 | - | 1 | - |
| high | 1.57  (0.89 – 2.79) | 0.121 | 1.78  (0.92 – 3.43) | 0.085 | 1.95  (1.04 – 3.65) | 0.037 |
|  |  |  |  |  |  |  |

Results from the Cox proportional hazards model. OAS (overall survival), DSS (disease-specific survival), DFS (disease-free survival).

**Supplement Table 2.** Uni- and multivariate 3-year survival analysis of oral cavity squamous cell carcinoma (OCSCC) patients. Hazard ratios (HR), 95% confidence intervals (CI), and *p* values were reported. Tobacco use was defined as daily smoking at the time of diagnosis.

|  |  |  | **Univariate** |  |  |  |  |  | **Multivariate** |  |  |  |
| --- | --- | --- | --- | --- | --- | --- | --- | --- | --- | --- | --- | --- |
|  | **3-year OAS** |  | **3-year DSS** |  | **3-year DFS** |  | **3-year OAS** |  | **3-year DSS** |  | **3-year DFS** |  |
|  | HR (95% CI) | *P* value | HR (95% CI) | *P* value | HR (95% CI) | *P* value | HR (95% CI) | *P* value | HR (95% CI) | *P* value | HR (95% CI) | *P* value |
| **Age**  < 65  ≥ 65 | 1  1.76  (1.14 – 2.72) | -  0.011 | 1  1.43  (0.87 – 2.34) | -  0.161 | 1  1.54  (1.01 – 2.33) | -  0.043 | 1  1.89  (1.22 – 2.94) | -  0.005 | 1  1.56  (0.95 – 2.61) | -  0.077 | 1  1.63  (1.07 – 2.48) | -  0.024 |
| **T-class** |  |  |  |  |  |  |  |  |  |  |  |  |
| T1-2 | 1 | - | 1 | - | 1 | - | 1 | - | 1 | - | 1 | - |
| T3-4 | 4.48  (3.01 – 6.66) | <0.001 | 5.19  (3.22 – 8.36) | <0.001 | 3.35  (2.27 – 4.93) | <0.001 | 4.48  (2.81 – 7.14) | < 0.001 | 4.58  (2.62 – 8.00) | <0.001 | 2.96  (1.88 – 4.66) | <0.001 |
| **N-class** |  |  |  |  |  |  |  |  |  |  |  |  |
| N0 | 1 | - | 1 | - | 1 | - | 1 | - | 1 | - | 1 | - |
| N+ | 2.26  (1.53 – 3.34) | < 0.001 | 2.84  (1.79 – 4.51) | <0.001 | 2.29  (1.56 – 3.36) | <0.001 | 1.25  (0.79 – 1.98) | 0.339 | 1.50  (0.87 – 2.59) | 0.141 | 1.55  (0.99 – 2.43) | 0.054 |
| **Tobacco use** |  |  |  |  |  |  |  |  |  |  |  |  |
| No | 1 | - | 1 | - | 1 | - | not included |  | not included |  | not included |  |
| Yes | 1.14  (0.77 – 1.70) | 0.507 | 1.21  (0.76 – 1.93) | 0.419 | 0.90  (0.60 – 1.40) | 0.602 |  |  |  |  |  |  |
| **xCT** |  |  |  |  |  |  |  |  |  |  |  |  |
| low | 1 | - | 1 | - | 1 | - | 1 | - | 1 | - | 1 | - |
| high | 0.68  (0.46 – 1.02) | 0.059 | 0.74  (0.46 – 1.18) | 0.208 | 0.74  (0.50 – 1.07) | 0.123 | 0.57  (0.38 – 0.86) | 0.007 | 0.63  (0.39 – 1.01) | 0.054 | 0.63  (0.42 – 0.93) | 0.022 |
|  |  |  |  |  |  |  |  |  |  |  |  |  |

Results from the Cox proportional hazards model. OAS (overall survival), DSS (disease-specific survival), DFS (disease-free survival).

**Supplement Table 3.** Uni- and multivariate 3-year survival analysis of laryngeal squamous cell carcinoma patients. Hazard ratios (HR), 95% confidence intervals (CI), and p values were reported. Tobacco use was defined as daily smoking at the time of diagnosis.

|  |  |  | **Univariate** |  |  |  |  |  | **Multivariate** |  |  |  |
| --- | --- | --- | --- | --- | --- | --- | --- | --- | --- | --- | --- | --- |
|  | **3-year OAS** |  | **3-year DSS** |  | **3-year DFS** |  | **3-year OAS** |  | **3-year DSS** |  | **3-year DFS** |  |
|  | HR (95% CI) | *P* value | HR (95% CI) | *P* value | HR (95% CI) | *P* value | HR (95% CI) | *P* value | HR (95% CI) | *P* value | HR (95% CI) | *P* value |
| **Age**  < 65  ≥ 65 | 1  0.87  (0.45 – 1.69) | -  0.677 | 1  0.60  (0.24 – 1.53) | -  0.284 | 1  1.00  (0.51 – 1.98) | -  0.999 | not included |  | not included |  | not included |  |
| **T-class** |  |  |  |  |  |  |  |  |  |  |  |  |
| T1-2 | 1 | - | 1 | - | 1 | - | 1 | - | 1 | - | 1 | - |
| T3-4 | 3.33  (1.69 – 6.58) | < 0.001 | 6.46  (2.32 – 18.02) | < 0.001 | 2.76  (1.38 – 5.52) | 0.004 | 2.69  (1.32 – 5.48) | 0.006 | 4.89  (1.67 – 14.12) | 0.004 | 2.34  (1.14 – 4.82) | 0.021 |
| **N-class** |  |  |  |  |  |  |  |  |  |  |  |  |
| N0 | 1 | - | 1 | - | 1 | - | 1 | - | 1 | - | 1 | - |
| N+ | 3.46  (1.75 – 6.28) | <0.001 | 7.37  (2.95 – 18.39) | <0.001 | 2.82  (1.39 – 5.75) | 0.004 | 2.61  (1.26 – 5.41) | 0.010 | 5.83  (2.15 – 15.81) | <0.001 | 2.28  (1.06 – 4.92) | 0.035 |
| **Tobacco use** |  |  |  |  |  |  |  |  |  |  |  |  |
| No | 1 | - | 1 | - | 1 | - |  |  |  |  |  |  |
| Yes | 1.21  (0.57 – 2.58) | 0.624 | 1.18  (0.43 – 3.29) | 0.746 | 0.93  (0.44 – 1.95) | 0.840 | not included |  | not included |  | not included |  |
| **xCT** |  |  |  |  |  |  |  |  |  |  |  |  |
| low | 1 | - | 1 | - | 1 | - | 1 | - | 1 | - | 1 | - |
| high | 1.44  (0.73 – 2.83) | 0.292 | 1.34  (0.54 – 3.34) | 0.526 | 1.25  (0.63 – 2.50) | 0.521 | 1.05  (0.52 – 2.12) | 0.902 | 0.67  (0.25 – 1.81) | 0.428 | 0.94  (0.45 – 1.94) | 0.859 |
|  |  |  |  |  |  |  |  |  |  |  |  |  |

Results from the Cox proportional hazards model. OAS (overall survival), DSS (disease-specific survival), DFS (disease-free survival).

**Supplement Table 4.** Multivariate 3-year survival analysis of p16 in OPSCC patients. Hazard ratios (HR), 95% confidence intervals (CI), and p values were reported. Tobacco use was defined as daily smoking at the time of diagnosis.

|  | **3-year OAS** |  | **3-year DSS** |  | **3-year DFS** |  |
| --- | --- | --- | --- | --- | --- | --- |
|  | HR (95% CI) | *P* value | HR (95% CI) | *P* value | HR (95% CI) | *P* value |
| **Age**  < 65  ≥ 65 | 1  1.44  (0.78 – 2.66) | -  0.243 | 1  1.76  (0.91 – 3.40) | -  0.092 | 1  2.03  (1.12 – 3.70) | -  0.021 |
| **T-class** |  |  |  |  |  |  |
| T1-2 | 1 | - | 1 | - | 1 | - |
| T3-4 | 2.60  (1.38 – 4.87) | 0.003 | 2.99  (1.46 – 6.12) | 0.003 | 2.34  (1.25 – 4.38) | 0.008 |
| **N-class** |  |  |  |  |  |  |
| N0 | 1 | - | 1 | - | 1 | - |
| N+ | 1.18  (0.64 – 2.19) | 0.593 | 1.74  (0.84 – 3.62) | 0.139 | 2.02  (1.01 – 4.03) | 0.046 |
| **Tobacco use** |  |  |  |  |  |  |
| No | 1 | - | 1 | - | 1 | - |
| Yes | 2.28  (1.03 – 5.01) | 0.041 | 2.28  (0.96 – 5.42) | 0.062 | 2.18  (1.02 – 4.64) | 0.045 |
| **p16** |  |  |  |  |  |  |
| positive | 1 | - | 1 | - | 1 | - |
| negative | 1.99  (0.95 – 4.14) | 0.067 | 1.85  (0.83 – 4.11) | 0.131 | 1.60  (0.78 – 3.31) | 0.203 |
|  |  |  |  |  |  |  |

Results from the Cox proportional hazards model. OAS (overall survival), DSS (disease-specific survival), DFS (disease-free survival).

**References:**

1. Linher-Melville K, Sharma M, Nakhla P, et al. Inhibiting STAT3 in a murine model of human breast cancer-induced bone pain delays the onset of nociception. *Mol Pain*. 2019;15.

2. Menschikowski M, Platzbecker U, Hagelgans A, et al. Aberrant methylation of the M-type phospholipase A(2) receptor gene in leukemic cells. *BMC Cancer*. 2012;12.

3. Mylly M, Nissi L, Huusko T, et al. Epidemiological Study of p16 Incidence in Head and Neck Squamous Cell Carcinoma 2005&ndash;2015 in a Representative Northern European Population. *Cancers 2022, Vol 14, Page 5717*. 2022;14(22):5717.
